# The genus *Arachis*: an excellent resource for studies on differential gene expression for stress tolerance

**DOI:** 10.3389/fpls.2023.1275854

**Published:** 2023-10-30

**Authors:** Dilip Kumar, Pulugurtha Bharadwaja Kirti

**Affiliations:** ^1^ Department of Microbial Genetics and Gene Expression, Institute of Microbiology of the Czech Academy of Sciences, Prague, Czechia; ^2^ Agri Biotech Foundation, Professor Jayashankar Telangana State (PJTS) Agricultural University, Hyderabad, Telangana, India

**Keywords:** peanut (*Arachis hypogaea*), wild Arachis species, differential gene expression (DEGs), RNA-seq, biotic stress, abiotic stress

## Abstract

Peanut *Arachis hypogaea* is a segmental allotetraploid in the section *Arachis* of the genus *Arachis* along with the Section *Rhizomataceae.* Section *Arachis* has several diploid species along with *Arachis hypogaea* and *A. monticola*. The section *Rhizomataceae* comprises polyploid species. Several species in the genus are highly tolerant to biotic and abiotic stresses and provide excellent sets of genotypes for studies on differential gene expression. Though there were several studies in this direction, more studies are needed to identify more and more gene combinations. Next generation RNA-seq based differential gene expression study is a powerful tool to identify the genes and regulatory pathways involved in stress tolerance. Transcriptomic and proteomic study of peanut plants under biotic stresses reveals a number of differentially expressed genes such as *R* genes (NBS-LRR, LRR-RLK, protein kinases, MAP kinases), pathogenesis related proteins (PR1, PR2, PR5, PR10) and defense related genes (defensin, F-box, glutathione S-transferase) that are the most consistently expressed genes throughout the studies reported so far. In most of the studies on biotic stress induction, the differentially expressed genes involved in the process with enriched pathways showed plant-pathogen interactions, phenylpropanoid biosynthesis, defense and signal transduction. Differential gene expression studies in response to abiotic stresses, reported the most commonly expressed genes are transcription factors (MYB, WRKY, NAC, bZIP, bHLH, AP2/ERF), LEA proteins, chitinase, aquaporins, F-box, cytochrome p450 and ROS scavenging enzymes. These differentially expressed genes are in enriched pathways of transcription regulation, starch and sucrose metabolism, signal transduction and biosynthesis of unsaturated fatty acids. These identified differentially expressed genes provide a better understanding of the resistance/tolerance mechanism, and the genes for manipulating biotic and abiotic stress tolerance in peanut and other crop plants. There are a number of differentially expressed genes during biotic and abiotic stresses were successfully characterized in peanut or model plants (tobacco or *Arabidopsis*) by genetic manipulation to develop stress tolerance plants, which have been detailed out in this review and more concerted studies are needed to identify more and more gene/gene combinations.

## Introduction

Peanut (*Arachis hypogaea* L.) is one of the most important legume crops economically worldwide and its seed a source of high quality edible oil, proteins, minerals and vitamins. It is widely cultivated across developing countries from semi-arid tropics to subtropical regions (Shoba et al., 2012). China and India contribute the highest global share of 34% and 19% of world peanut production ranking 1^st^ and 2^nd^ respectively based on FAOSTAT data for the year 2020 (FAOSTAT, 2020). Production and yield of peanut are severely affected by several biotic and abiotic factors. The biological stress factors comprise diseases caused by fungi, bacteria, viruses, nematodes and insect pests, whereas the abiotic constraints include drought, salinity, water logging, temperature and light. As the crop is dependent on seasonal rainfall, its productivity is dependent on the adverse effects of the environment in the form of stresses, both biotic and abiotic. Such stresses greatly decrease crop growth, yield and productivity.

The responses of plants to overcome these stress conditions comprise a number of cellular and molecular mechanisms. Developing biotic and abiotic resistant peanut cultivars by conventional breeding techniques has been limited due to narrow usable genetic variability and linkage drag in interspecifc crosses that transfer the desired genes along with unnecessary genes from wild to the cultivated peanuts, which results in introgressed lines that cannot be incorporated in peanut varietal development programs. Cultivated peanut genome (2.7 Gb) is quite large in size (Bertioli et al., 2019) in contrast to other plant models, *Arabidopsis* (135 Mb), rice (430 Mb), *Medicago* (904 Mb) and soybean (1.1 Gb). Large genome size and polyploidy nature (Anderson et al., 2005) hamper crop improvement and genetics research in peanut like studying gene function, metabolic responses, regulatory pathways that are activated under stress conditions, and the identification of resistance genes and potential genes that impart tolerance to various stresses. Despite transcriptome analyses, there are only a few reports on the existence of suitable genes for stress tolerance in the cultivated accessions of peanut. However, wild accessions in the Genus *Arachis* are shown to possess genes for stress tolerance, which can be subjected to cloning through appropriate genomic approaches.

The genus *Arachis* comprises many wild species at different ploidy levels that exhibit resistance/tolerance to several biotic and abiotic stresses, which makes it a rich resource of suitable genes for commercial applications. Some wild species were deployed in the experiments with the aim of transferring the genes for tolerance/resistance to different stresses from the related wild species to the cultivated peanut genotypes in crop improvement programs (Wynne et al., 1991; Singh et al., 1997). However, these efforts did not result in expected improvements in various commercial traits of peanut as the gene introgression strategies further resulted in a linkage drag that transfers potentially important genes along with the undesirable genes into the cultivated peanut background. Better strategy to develop resistance/tolerance in peanut is to identify and clone the homologs of the resistance genes in the related species and deploy them in peanut improvement programs. Several diploid wild species of the genus *Arachis*, Viz., *A. diogoi, A. stenosperma, A. cardenasii*, *A. duranensis* etc. show very high levels of resistance/tolerance to biotic and abiotic stresses (Pande and Rao, 2001). These species will be suitable targets for studying the differences at molecular level that determine resistance or susceptibility to stress conditions. The best strategy to avoid significant economic loss is to identify resistance genes under stress conditions by differential gene expression analyses and development of stress tolerance cultivars.

Several methods such as RFLP, AFLP, SSH, Microarray and RNA-seq have been reported to estimate transcript expression levels between the samples (Vos et al., 1995; Payton et al., 2009; Katz et al., 2010; Li et al., 2010; Guo et al., 2011; Turro et al., 2011; Rathod et al., 2020). RNA-seq based differential gene expression (DGE) analysis is a relatively recent method to analyze gene expression within a transcriptome and the interpretation of differences in enrichment of desirable gene transcripts, where a large number of genes are differentially expressed between the samples (Conesa et al., 2016). Furthermore, RNA-seq data analysis tools can generate a list of genes that are differentially expressed between two samples of data sets.

Next-generation RNA sequencing (RNA-seq) is well-established and versatile technique with application to detects the enriched sequences in specific tissues at specific time points and used to characterize differential gene expression of plant responses to biotic and abiotic stresses (Martin et al., 2013; Wang et al., 2018; Ali et al., 2022). To investigate genome function and stress related pathways, the transcriptome analysis is a robust and highly efficient method (Mia et al., 2020; Gangurde et al., 2021). Several studies have been carried out using differential gene expression (transcriptome) analysis to identify genes and pathways for biotic and abiotic stresses in peanut (Brasileiro et al., 2015; Kumar and Kirti, 2015b; Shen et al., 2015; Han et al., 2017; Cui et al., 2018; Zhao et al., 2018; Jiang et al., 2021; Cui et al., 2022). Differentially expressed genes and their further characterization will be reviewed with emphasis on genes involved in biotic and abiotic stress tolerance in peanut in this communication.

## Biotic stresses

**Figure 1:**
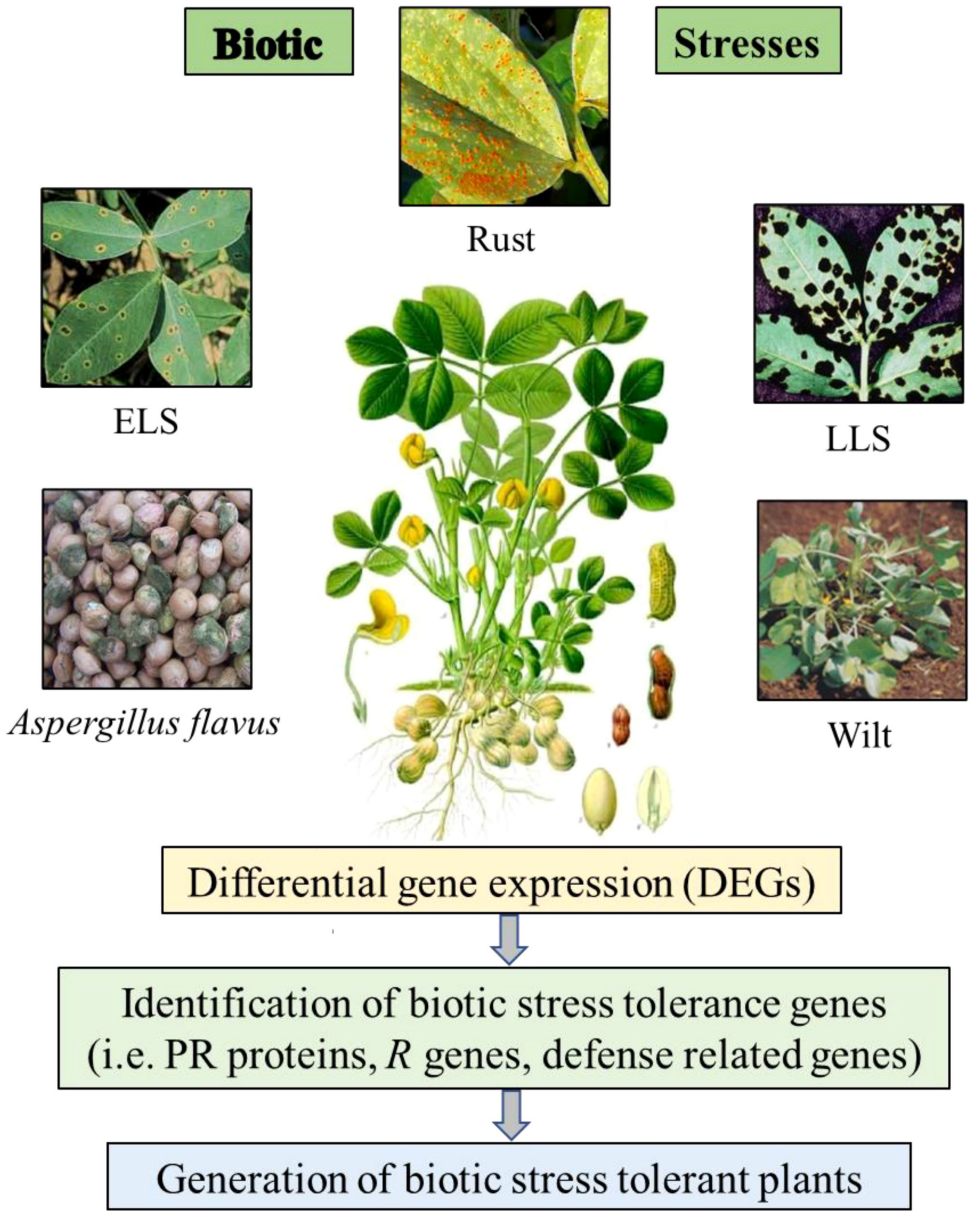
Schematic visualization of the differential gene expression (DEGs) study on peanut plants under biotic stress conditions.

Several biological stresses are known to limit peanut productivity and these factors comprise several diseases including fungal disease like early leaf spot (ELS), late leaf spot (LLS), rust, aflatoxin contamination and bacterial wilt disease by *Ralstonia solanacearum* (**Figure 1**), which are global constraints against peanut production (Subrahmanyam et al., 1984). To manage these various biotic stresses in peanut, it is necessary to study molecular machanisms involved during stress conditions and further implementaion of the outcome for improving quality and yield would help in developing biotic stress resistant cultivars. There are some reports of transcriptomic and proteomic studies in peanut to identify differentially expressed genes and proteins under biotic stress conditions and further characterization of candidate tolerance gene to develop biotic stress resistance plants.

## Early leaf spot disease

Foliar diseases of peanut have worldwide impact on yield and quality. Early leaf spot (ELS) and late leaf spot (LLS) diseases also collectively known as ‘Tikka’ disease caused by *Cercospora arachidicola* [Hori] and *Phaeosariopsis personata* [Berk & M. A. Curtis] respectively, are the major foliar fungal diseases in peanut, which cause complete defoliation of leaves leading to significant losses in plant productivity upto 50 to 70% (Subrahmanyam et al., 1984; Tshilenge-Lukanda et al., 2012). Early leaf spot symptoms are generally exhibited as brown lesions surrounded by a yellow halo on the upper surface of leaves (McDonald et al., 1985). Advanced technology like transcriptomics or RNA-seq study provides better understanding of gene expression upon pathogen invasion without prior knowledge on the genomes of the corresponding plants (Kawahara et al., 2012). Upon pathogen attack, plant elicits defense mechanisms that tend to neutralize such invasion by activating metabolic pathways and expression of defense related genes (Kumar and Kirti, 2015b). Candidate genes responsible for plant defense mechanism can be identified by differential genes expression study. However, differential gene expression analysis of resistance to early leaf spot disease in peanut has been limited. Recently, Rathod et al. (2020) analyzed the differentially expressed genes (DEGs) in resistant and susceptible peanut varieties upon infection with *Cercospora arachidicola*. They found an upregulation of defense related genes like PR protein genes, disease resistance response protein, polygalacturonase, a leucine-rich repeat (LRR) protein, transcription factors (WRKY, myc), peroxidases and genes related to secondary metabolites in the resistant variety upon pathogen invasion, while there was downregulation of genes including F-box, cytochrome p450, LRR protein kinase and terpene synthase that are associated with several biological processes in the susceptible variety. There is another recent report of DEGs of resistant and susceptible peanut cultivars to early leaf spot infection, which revealed the expression of resistance associated genes like CC-NB-LRR (NLR) type resistance gene, Phytoalexin deficient 4 (PAD 4) and polyphenol oxidase (PPO) that play important roles in mediating early leaf spot resistance (Gong et al., 2020). Identification of disease resistance trait linked genes through genome wide analysis by RNA-seq technology can play a major role in the identification of suitable candidate genes, whose deployment would result in the development of resistant peanut cultivars.

## Late leaf spot disease

Late leaf spot disease caused by *Pheaosariopsis personata* (*Cercospora personat*a) is the most devastating disease in peanut and can lead to yield losses up to 70% under favorable conditions (Subrahmanyam et al., 1989; Grichar et al., 1998). Late leaf spot symptoms exhibit dark brown or black lesions on the lower side of infected leaves (Tshilenge-Lukanda et al., 2012). Effective control of leaf spot diseases is to use fungicides that are not cost effective and could cause environmental pollution (Miller et al., 1990). Therefore, a better approach to improve resistance is to identify disease resistance gene homologs from a resistant peanut variety or wild relatives and utilize these resistance sources to develop resistant peanut cultivars. A number of diploid wild species like *A. diogoi, A. stenosperma, A. cardenasii*, *A. duranensis* exhibit resistance to fungal pathogens including the fungus *Phaeoisariopsis personata* that causes late leaf spot in peanut, which can be cloned through genomic approaches (Pande and Rao, 2001.

There are several methods available to study differential gene expression during plant-pathogen interactions in peanut, such as Genefishing DEG kit, suppression subtractive hybridization (SSH), cDNA-AFLP and cDNA-microarray techniques (Nobile et al., 2008; Guo et al., 2011; Kumar and Kirti, 2011; Kumar and Kirti, 2015b). These are simple and effective methods in the identification of DEGs as they are comparatively inexpensive in relation to the highly efficient transcriptomic RNA-seq based methods, which are expensive and need a lot of knowledge in Bioinformatic tools. In the absence of sufficient funding and experience in Bioinformatic tools, the earlier mentioned old methods allow one to embark on a differential gene expression studies. At present, the next-generation RNA-sequencing (RNA-seq) is an established and versatile platform with application in the quantification of gene expression in gene pools of many biological samples that can be exploited (Martin et al., 2013). Luo et al. (2005) identified several differentially expressed genes in resistant and susceptible peanut genotpyes using a cDNA microarray, but were unable to detect a resistant gene specific to late leaf spot infection. There is another gene expression study using suppression subtractive hybridization (SSH) technique, which identified a number of genes involved in defense signaling pathway, phenylpropanoid pathway, transcription factors and the most significantly upregulated gene corresponds to a novel *O*′-methyltransferase (Nobile et al., 2008). In an earlier study, treated leaf material of *Arachis diogoi* was analyzed for identifying differential gene expression using a Genefishing DEG kit after treatment with the late leaf spot causing pathogen and this study identified several defense related genes and genes related to phenylpropanoid pathway (Kumar and Kirti, 2011). Kumar and Kirti (2015b) also investigated the molecular responses of the wild peanut *Arachis diogoi* challenged with the late leaf spot pathogen using cDNA-AFLP and identified several differentially expressed genes (DEGs). Furthermore, they identified TDFs (transcript derived fragments) that are associated with defense, signal transduction and metabolism, and further reported several genes for proteins involved in hypersensitive cell death, cell wall fortification and defense mechanism. Differential gene expression analysis in wild type and mutant peanut cultivar against late leaf spot pathogen was also reported by Han et al. (2017), who reported a significant up-regulation of pathogenesis-related (PR) proteins, WRKY transcription factors and down-regulation of genes related to photosynthesis in susceptible genotype to cope up with stress factors. There is a report on gene locus based sequencing analysis to identify resistance genes for late leaf spot disease in a peanut cultivar by using double-digest restriction site associated DNA sequencing (ddRAD-Seq) technique, which is based on next-generation sequencing (NGS) for genotyping analysis and this study reported on the identification of four candidate genes (LRR and NB-ARC domain disease resistance protein, TIR-NBS-LRR disease resistance protein, MLO like protein) for late leaf spot disease (Shirasawa et al., 2018).

Resistance gene analogs (RGA), as potential resistance (R) genes play important roles in recognition and activation of disease resistance responses and the identification of putative peanut *R*-genes (381) associated with leaf spot disease was predicted by a RGA-PCR based technique (Dang et al., 2019). Further, the same group studied *R*-gene expression that is associated with leaf spot resistant peanut genotype by undertaking a differential gene expression study, which found that a majority of the genes are receptor like kinases (RLKs), receptor like proteins (RLPs) and receptor like cytoplasmic kinases (RLCKs), which co-ordinate and initiate protection responses against the invading pathogen (Dang et al., 2021). Recently, a comparative transcriptome analysis of resistant and susceptible peanut genotypes for late leaf spot disease has identified candidate genes for late leaf spot resistance. The authors have found differential upregulation of a putative disease resistance gene RPP-13 like, NBS-LRR protein, MAPK kinase, WRKY transcription factor and PR proteins in the resistant genotype. Furthermore, they identified the upregulation of tetratricopeptide repeats (TPR), pentatricopeptide repeat (PPR), chitinases, glutathione S-transferases, purple acid phosphatases in resistant genotypes, which also reported that a MLO-like proteins, Ubiquitin protein ligase and metal transport proteins that were upregulated in the susceptible genotypes. However, important pathways like antibiotic biosynthesis, phenylpropanoid biosynthesis and flavonoid biosynthesis were triggered in both genotypes in response to late leaf spot infection (Gangurde et al., 2021).

## Rust disease

Rust is another serious foliar disease of peanut caused by *Puccinia arachidis* that often occurs along with leaf spot disease also because of its rain fed nature, which leads to further yield losses. There are several studies on quantitative trait locus (QTL) in cultivated peanut to identify molecular marker or genetic map for rust resistance (Khedikar et al., 2010; Kolekar et al., 2016; Pandey et al., 2017; Mondal and Badigannavar, 2018), but there were no reports of differential gene expression studies on this pathogen attack, which can shed light on resistance genes involved during molecular interaction between peanut plant and respective fungal pathogen *Puccinia arachidis*. There was a sequencing analysis of genetic loci to identify resistance genes for rust disease in peanut cultivar by using double-digest restriction site associated DNA sequencing (ddRAD-Seq) technique, which is based on next-generation sequencing (NGS) for genotyping and this resulted in the identification of six candidate genes for rust resistance (Shirasawa et al., 2018). Recently, Rathod et al. (2020) have studied differential gene expression in resistant and susceptible genotypes of peanut for leaf rust infection and identified differentially expressed genes responsible for defense against rust disease in peanut, They reported altered metabolic pathways and defense related genes during this biotic stress. Further, differential expression of genes upregulated such as pathogenesis-related (PR) proteins, thaumatin like protein, polygalacturonase, ethylene-responsive factor, MLO-like protein and F-box protein in the resistant genotype, while genes like β-glucosidase, transcription factors (WRKY, MYB) and caffeate *O*-methyltransferase were downreglated in the susceptible genotype. It was also reported that several defense related genes such as cytochrome p450, chitinase, glutathion S-transferase and TIR-NBS-LRR (R-gene) protein were significantly upregulated in the resistant genotype revealing their important roles in plant defense mechanism. These findings will be helpful in understanding the molecular mechanism of peanut plant defense against the rust pathogen and may assist the breeders in the development of resistant varieties through molecular approaches.

## *Aspergillus flavus* (aflatoxin) infection

*Aspergillus flavus* is an opportunistic saprophytic fungal pathogen that infects a number of seed crops (maize, peanut, rice, cotton etc.) (Tumukunde et al., 2020) and produces highly toxic secondary metabolites called aflatoxins. Due to their toxicity, consumption of aflatoxin contaminated food can cause teratogenic and carcinogenic effects in animals and humans (Liang et al., 2006; Wang et al., 2010). Peanut kernels are contaminated by *Aspergillus flavus* during pre- and post-harvest conditions (Guo et al., 2011) that causes serious concerns for food safety. To date, not much information is available on the identification of resistance genes in peanut for resisting *Aspergillus flavus* infection using molecular techniques like differential gene expression analysis. Guo et al. (2011) identified *Aspergillus* resistance genes that were upregulated in a resistant variety of peanut in comparison to a susceptible cultivar in a large scale analysis using ESTs (expressed sequence tags) and microarray technology. Furthermore, they identified sixty two genes in the resistant cultivar that were upregulated in response to *Aspergillus* infection including defense related genes like PR10 protein, defensin, calmodulin, metallothionein like protein, Cu/Zn superoxide dismutase etc. and a large number of hypothetical proteins as the complete genome sequence was not available at that point of time. A similar study has reported the identification of *Aspergillus* resistance genes in peanut cultivars by comparing transcriptome profiles in resistant and susceptible peanut genotypes using microarray technique (Wang et al., 2012). Their results showed that the differentially expressed genes related to defense responses, oxidation-reduction, signaling, proteolysis metabolism, oligopeptide/transmembrane transport and carbohydrate metabolism were significantly expressed in the resistant cultivar in response to *A. flavus* infection. A comprehensive study of resistant and susceptible peanut transcriptome in response to *A. flavus* has been reported using RNA-seq technology (Wang et al., 2016b). This group has identified crucial defense related genes, like NB-LRR proteins, LRR receptor-like kinases, mitogen-activated protein kinase, pathogenesis-related proteins, polygalacturonase inhibitor proteins, transcription factors and other phenylpropanoid pathway related genes that were differentially expressed in the resistant genotype and this information might contribute toward the development of resistance to aflatoxin production in peanut. Most recently, Cui et al. (2022) analyzed the transcriptomes of resistant and susceptible peanut genotypes in response to *A. flavus* at different time points using weighted gene co-expression network analysis (WGCNA) and identified hub genes positively associated with resistance to *A. flavus* in peanut. Their analysis also revealed that upregulation of genes encoding pathogenesis-related protein (PR10), MAPK kinase, 1-aminocyclopropane-1-carboxylate oxidase (ACO1), a serine/threonine kinase, cytochrome P450, pectin esterase, SNARE protein SYP121, pentatricopeptide repeat (PPR) protein and disease resistance response proteins in the esistant peanut cultivar that play major roles in resistance to infection from *A. flavus*. These studies provide new insights into the molecular mechanism of peanut defense against aflatoxin contamination and further the safety and management of peanut products for human consumption.

## Wilt disease

Bacterial wilt (caused by *Ralstonia solanacearum*) is the most devastating soil borne disease in peanut (*Arachis hypogaea* L.) leading to significant yield losses because of reduced plant stand on the field at the seedling stage of the crop. Molecular mechanism of peanut response to *R. solanacearum* remains unnknown and needs to be studied in detail. In an attempt to explore the molecular mechanism of bacterial wilt resistance in peanut using Genefishing DEG kit, several differentially expressed candidate genes encoding a cyclophilin, ADP-ribosylation factor, antibacterial peptide and disease resistance response proteins were identified by studying the differences in gene expression between inoculated and control peanut seeds (Ding et al., 2012). In another attempt, suppression subtractive hybridization (SSH) and microarray hybridization were used to detect differentially expressed genes (DEGs) in the roots of wild peanut upon *R. solanacearum* inoculation and this study reported deferentially expressed genes that are involved in the biosynthesis of phytoalexins, which might play crucial role in resistance to wilt disease (Chen et al., 2014b). In a root transcriptome analysis of resistant and susceptible peanut genotypes after infection with *R. solanacearum*, KEGG analysis showed that the primary metabolism got inhibited more in the resistant genotype at an early point of inoculation exhibiting similar response like the susceptible genotype. Moreover, defense related genes like *R* gene, cell wall genes, LRR-RLK protein etc. were differently expressed between both the genotypes (Chen et al., 2014c). Yang et al. (2022) analyzed differential gene expression in leaves of resistant and susceptible peanut genotypes infected with *R*. *solanacearum* by cutting leaf method and classified differentially expressed genes (DEGs) into six groups as resistant/susceptible response genes, which included PAMPs induced resistant/susceptible response genes and type III effectors (T3Es) induced resistant/susceptible response genes. Further more, KEGG enrichment pathway analysis of differentially expressed genes showed that MAPK signaling, plant-pathogen interaction, and plant hormone signal transduction pathways were upregulated. WRKY Transcription factors play an important role in plant disease resistance. Differential gene expression analysis of WRKY genes in cultivated peanut displayed different expression patterns in resistant and sensitive peanut cultivars infected with *R*. *solanacearum*. The identification of candidate WRKY genes with possible role in peanut resistance to *R*. *solanacearum* infection may contribute in the improvement of a resistant peanut variety (Yan et al., 2022).

Transcriptomic and proteomic studies of peanut plants in response to biotic stresses, revealed a number of differentially expressed genes such as *R* genes (CC-NBS-LRR, TIR-NBS-LRR, LRR-RLK, protein kinases, MAP kinases), pathogenesis related proteins (PR1, PR2, PR5, PR10), defense related genes (defensin, F-box, polygalacturonase, cytochrome p450, pentatricopeptide repeat-containing protein (PPR), glutathione S-transferase) and the genes involved in phenylpropanoid pathway are the most commonely expressed ones (**Table 1**). These differentially expressed genes involved in the process or enriched pathways of plant-pathogen interactions included phenylpropanoid biosynthesis, secondary metabolism, defense and signal transduction. Some of the differentially expressed genes were selected for further characterization in peanut or model plants (tobacco or *Arabidopsis*) through genetic manipulation and successfully developed biotic stress resistant plants (**Table 2**).

**Table 1:** Some important differentially expressed peanut genes and enriched pathways or genes involved in process during various biotic stress conditions.

Stresses	Technique used	Some important differentially expressed genes (DEGs)	Enriched pathways/genes involved in	References
ELS	RNA-seq	cell wall associated protein genes (polygalacturonase, PR proteins (thaumatin), chitinase & pectin esterase), *R* genes (NBS-LRR), cytochrome p450, glutathione s-transferase, heat-shock protein40, transcription factors (WRKY & bHLH), ascorbate & glutathione peroxidases,	plant-pathogen interaction, MAPK signaling, biosynthesis of secondary metabolites	Rathod et al. (2020)
	RNA-seq	*R* gene (CC-NBS-LRR), interleukin-1 receptor associated kinase, serine/threonine-protein kinase, pectin esterase, phytoalexin deficient 4 (PAD4), polyphenol oxidase, E3 ubiquitin-protein ligase, auxin responsive GH3 gene, ATP-dependent RNA helicase, nuclear pore complex protein	phenylpropanoid biosynthesis, plant-pathogen interaction, biosynthesis of secondary metabolites, MAPK signaling	Gong et al. (2020)
LLS	Microarray	caffeic acid O-methyltransferase, heat shock protein, LRR- protein kinase, cytochrome p450, transcription factors (bZIP, MYB, zinc finger), PR proteins, Bax inhibitor, gltathione S-transferase, superoxide dismutase, disease resistant related proteins	secondary metabolism, signaling components, transcription factors, defense response	Luo et al. (2005)
	SSH	serine/threonine protein kinase, cyclin-dependent kinases, diacylglycerol kinase, caffeic acid methyl transferase, O-methyltransferase, cinnamoyl-CoA reductase, putative leucine zipper protein, auxin-regulated protein, lipid transfer protein, heat-shock protein 80, senescence-associated protein	signal transduction, phenylpropanoid pathway, transcription factors, defense response	Nobile et al. (2008)
	Genefishing DEG	cinnamic acid 4-hydroxylase, phenylalanine ammonia lyase (PAL), cinnamyl alcohol dehydrogenase (CAD), CBL- interacting protein kinase, LRR protein kinase, cyclophilin, PR protein (TLP), glutathione S-transferase, cytochrome p450 monooxygenase, superoxide dismutase, papain-like cysteine proteinase	phenylpropanoid biosynthesis, plant-pathogen interaction, defense response	Kumar and Kirti (2011)
	cDNA-AFLP/2D	CC-NB-LRR type disease resistance protein, SGT1, thaumatin-like protein (TLP), defensin like protein, heat shock 70 kDa protein, dihydroflavonol-4-reductase, cytochrome p450 monooxygenase, LEA protein, vacuolar-processing enzyme, F-box protein, zinc finger protein, chaperone protein DnaJ-like, racGTPase, protein kinase	defense response, signal transduction, metabolism, transcription factors	Kumar and Kirti (2015b)
	RNA-seq	disease resistance genes (RPT-13 like, NBS-LRR), F-box proteins, defensins, tetratricopeptide reapeat (TPR), pentatricopeptide repeat (PPR), chitinases, glutathione S-transferase, PR proteins, chalcone synthase, lipid transfer protein, transcription factors (WRKY & MYB), MLO-like protein, ethylene responsive factor	antimicrobial biosynthesis, phenylpropanoid pathway, flavonoid biosynthesis	Gangurde et al. (2021)
Rust	RNA-seq	*R* genes (NBS-LRR), F-box, MLO-like, polygalactourinase, transcription factors (WRKY, MYB, bZIP), ethylene responsive factor, chitinase, cytochrome p450, glutathione S-transferase, pathogenesis related (PR) proteins, thaumatin like proteins	defense mechanism, plant-pathogen interaction, antimicrobial biosynthesis, signal transduction	Rathod et al. (2020)
Aflatoxin	Microarray	formamidase, transferase, NADP-dependent malic enzyme, defensin, TIR-NBS-LRR, brassinosteroid insensitive 1-associated receptor kinase, serine O-acetyltransferase, 3-ketoacyl-CoA synthase, pentatricopeptide repeat-containing protein (PPR)	oxidation-reduction, defense response and signaling, coenzyme-A biosynthesis, proteolysis metabolism	Wang et al. (2012)
	RNA-seq	phenylalanine ammonia-lyase (PAL), cinnamate 4-hydroxylase (C4H) and 4-coumarate CoA ligase (4CL), chalcone synthase (CHS), stilbene synthase (STS), NBS-LLR protein, polygalacturonase, mitogen-activated protein (MAP) kinase, LRR receptor-like kinase, transcription factors (WRKY, bZIP, ERF), pathogenesis related (PR) proteins (PR1, PR2, PR5, PR10)	phenylpropanoid biosynthesis, flavonoid biosynthesis, stilbenoid biosynthesis, defense related	(Wang et al., 2016b)
	RNA-seq	pathogenesis-related proteins (PR10), MAP kinase, serine/threonine protein kinase, cytochrome p450, pectinesterase, phosphatidylinositol transfer protein, aminocyclopropane-1-carboxylate oxidase (ACO1), pentatricopeptide repeat (PPR) protein, SNARE protein SYP121	plant-pathogen interaction, phenylpropanoid biosynthesis, brassinosteroid biosynthesis, defense and signal transduction	(Cui et al., 2022)
Wilt	SSH and microarray	genes involved in the biosynthesis of sesquiterpenoids and triterpenoids, chalcone synthase (CHS), stilbene synthase, ethylene responsive factor (ERF), resveratrol synthase (RS), pathogenesis related (PR10) protein, MAPK protein kinase	carbohydrate metabolism, fatty acid metabolism, oxidative phosphorylation, defense and signal transduction	Chen et al. (2014b)
	RNA-seq	pyruvate dehydrogenase, isocitrate dehydrogenase, fructosidase, pectinesterase, polygalacturonase, phosphoglucose isomerase, ADP-ribosylation factor, chalcone synthase, terpenoid synthase, PR proteins (PR1, PR2, PR5, PR10), zinc finger, disease resistance protein, NBS-LRR, LRR-RLK, MAP kinase, transcription factors (ERF, WRKY, MYB)	carbohydrate metabolism, fatty acid metabolism, oxidative phosphorylation, defense and signal transduction	Chen et al. (2014c)
	RNA-seq	auxin-responsive genes, PR proteins (PR1, PR2, PR5), pectin esterase, polygalacturonase inhibitor, defensin, calmodulin-like proteins, calcium-dependent protein kinases, calcineurin B-like proteins, cytochrome p450, MAPK kinases, LRR kinase, zinc finger, WRKY transcription factor, chalcone and terpene synthase, isoflavone reductase, 2-hydroxyisoflavanone synthase, glutathione S-transferase, serine/threonine protein kinase	plant-pathogen interaction, signal transduction, MAPK signaling pathway, phenylpropanoid and flavonoid biosynthesis	Yang et al. (2022)

ELS, early leaf spot; LLS, late leaf spot; SSH, suppression subtractive hybridization.

**Table 2:** List of differentially expressed peanut genes used in enhanced biotic tolerance.

Gene	Characterized in	Stress tolerance	Identification method	Promoter used	References
*AdCyp*	tobacco	biotic stress	Genefishing DEG	CaMV35S	Kumar and Kirti (2011)
*AdRSZ21*	tobacco	biotic stress and HR-like cell death	Genefishing DEG	XVE (estradiol inducible promoter)	Kumar and Kirti (2012)
*AdSGT1*	peanut and tobacco	induced cell death and enhance disease resistance	cDNA-AFLP	CaMV35S	Kumar and Kirti (2015a)
*AdVPE*	tobacco	biotic stress	cDNA-AFLP	CaMV35S	Kumar et al. (2015)
*AdZADH2*	tobacco	biotic stress and HR-like cell death	cDNA-AFLP	XVE (estradiol inducible promoter)	Kumar et al. (2016)
*AhRRS5*	tobacco	biotic stress	Microarray	CaMV35S	Zhang et al. (2017)
*AhRLK1*	tobacco	biotic stress (wilt & Hypersensitive response)	Microarray	CaMV35S	Zhang et al. (2019)

## Transgenic plants reported for differentially expressed peanut genes for enhanced biotic stress tolerance

Several peanut genes, which were differentially expressed during biotic stress conditions were further characterized by transgenic approach. Presently, genetic engineering technique such as *Agrobacterium tumefaciens* mediated transformation is a powerful tool that can successfully incorporate genes controlling desirable traits into genomic DNA of peanut or model plants (*Arabidopsis* or tobacco) for functional characterization of genes that integrated into the corresponding genome. There are limited reports on differentially expressed peanut genes for further characterization using genetic engineering to develop stress tolerance plants. The transgenic plants that were developed for functional characterization of diffrentially expressed peanut genes are given in **Table 2**.

A *Cyclophillin* (*AdCyp*) gene that was differentially expressed in wild peanut *A. diogoi* during late leaf spot infection was incorporated into tobacco genome under a constitutive promoter through the *Agrobacterium* method and this resulted in enhanced resistance to *Ralstonia solanacearum* and reduced susceptibility toward *Phytophthora parasitica* var. *nicotianae*. Further, the resistance phenomenon was associated with the up-regualtion of various defense related genes (Kumar and Kirti, 2011). Similarly, *AdRSZ21* is a novel splicing factor gene that was identified in the differentially expressed genes upon pathogen challenge in wild peanut genotype. Its transient conditional expression in tobacco leaves resulted in HR-like cell death. Furthermore, the hypersensitive cell death induced by AdRSZ21 was associated with the upregulation of patatin-like protein gene and other defense related genes suggesting its crucial role in plant defense (Kumar and Kirti, 2012).

SGT1 is an essential signaling component in *R*-gene mediated resistance responses against various plant pathogens, which is widely conserved in eukaryotes and plays a major role in the resistance phenomenon against pathogens (Austin et al., 2002; Muskett and Parker, 2003; Liu et al., 2004). *SGT1* was differentially expressed in the resistant wild peanut upon challenge with the late leaf spot pathogen (Kumar and Kirti, 2015b). Overexpression of *AdSGT1* induced hypersensitive-like cell death in tobacco under transient conditional expression, and this conditional expression of *AdSGT1* was also associated with the upregulation of genes for proteins involved in hypersensitive responses. Moreover, consitutive expression of *AdSGT1* in cultivated peanut (susceptible) genotype enhanced resistance to late leaf spot pathogen and this expression was associated with the co-expression of resistance-related genes, CC-NB-LRR and some protein kinases, while heterologous expression in tobacco enhanced its resistance against *Phytophthora parasitica* var. *nicotianae*, *Alternaria alternata* var. *nicotianae* and *Rhizoctonia solani* (Kumar and Kirti, 2015a).

Vacuolar processing enzymes (VPEs) are cysteine proteases exhibiting caspase-1-like activity, which mediate cell death and upregulated during pathogen infections (Hatsugai et al., 2004; Kuroyanagi et al., 2005). *AdVPE* was found to be up-regulated in a differential gene expression study in resistant peanut genotype against late leaf spot pathogen. Transient conditional expression studies of *AdVPE* in tobacco leaves using agroinfiltration also resulted in hypersensitive response (HR) like cell death and positively regulated defense response genes. Furthermore, ectopic expression of *AdVPE* in tobacco resulted in enhanced resistance against *Phytophthora parasitica* var. *nicotianae*, *Alternaria alternata* var. *nicotianae* and *Rhizoctonia solani* (Kumar et al., 2015). A novel zinc-binding alcohol dehydrogenase 2 (*AdZADH2*) was also differentially upregulated in *Arachis diogoi*, a wild peanut upon challenge with the late leaf spot (LLS) pathogen. Transient over-expression of *AdZADH2* under an estradiol inducible promoter (XVE) exhibited hypersensitive response (HR)-like cell death in tobacco leaf and the cell death was associated with the upregulation of antioxidative enzymes such as SOD, CAT and APX and pathogenesis-related (PR) proteins (Kumar et al., 2016).

NBS-LRR proteins are the *R* gene products, which can directly or indirectly recognize pathogen effector proteins and induce signaling pathways for resistance against the impending pathogen (Cesari et al., 2013; Sohn et al., 2014). A novel peanut NBS-LRR resistant gene *AhRRS5* was differentially upregulated in response to *Ralstonia solanacearum* infection in both resistant and susceptible peanut cultivars in a microarray study. Transient overexpression of *AhRRS5* induced hypersensitive response in tobacco and overexpression of *AhRRS5* in tobacco significantly enhanced resistance to *R. solanacearum* along with increased expression of *NPR1* and *R* gene signal for defense (Zhang et al., 2017). Leucine-rich repeat receptor-like protein kinases (LRR-RLKs) are also involved in plant defense related disease resistance ((Godiard et al., 2003). A leucine-rich repeat receptor-like kinase gene *AhRLK1* was differentially expressed in a peanut cultivar after inoculation with *Ralstonia solanacearum* during a microarray study. Transient expression of *AhRLK1* resulted in hypersensitive response (HR) in *Nicotiana benthamiana* and *AhRLK1* overexpression in transgenic tobacco significantly enhanced resistance to *R. solanacearum* by triggering EDS1 and PAD4 in the *R* gene signaling pathway that possibly contributed to defense responses against the pathogen (Zhang et al., 2019).

## Abiotic stresses

**Figure 2:**
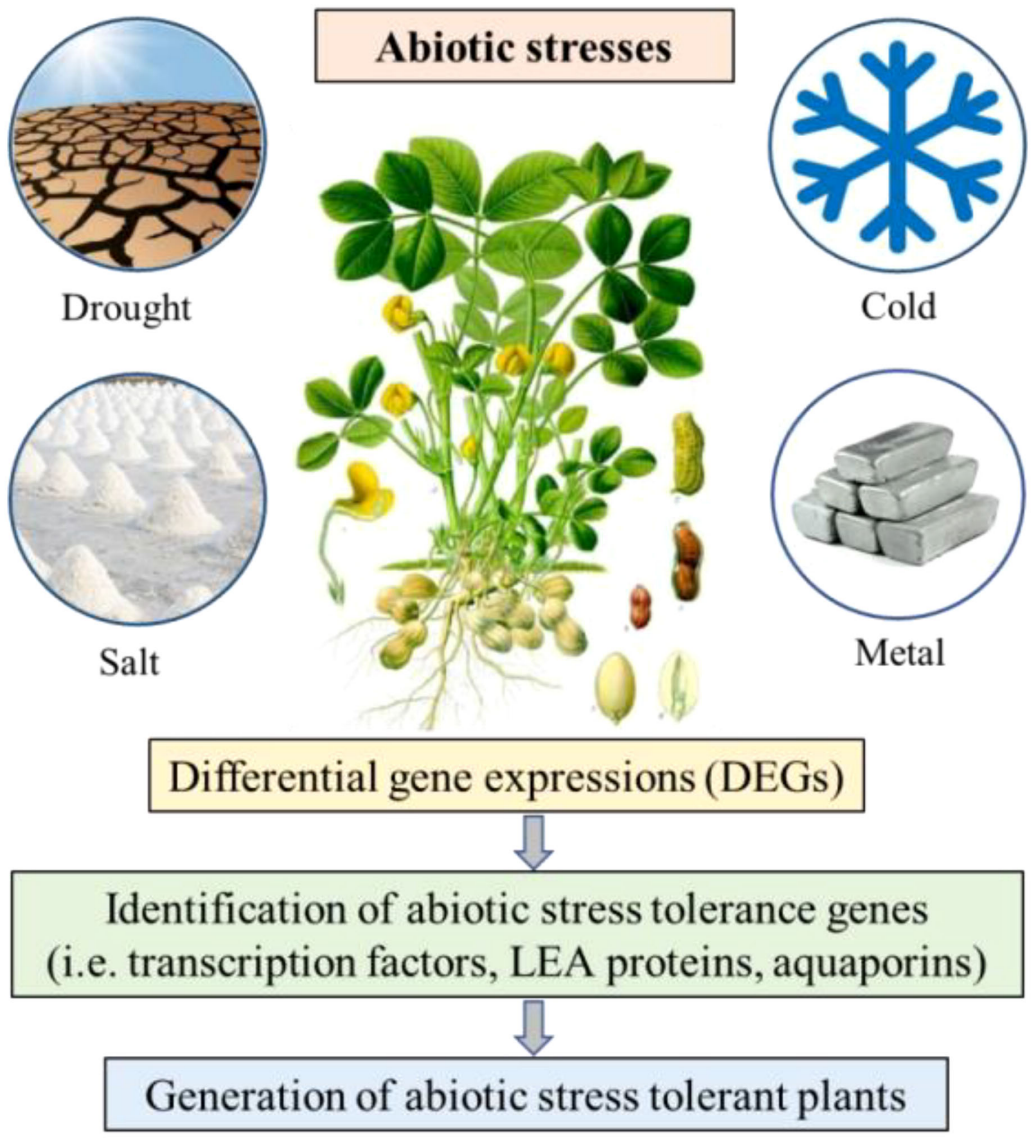
Schematic representation of the differential gene expression study on peanut under abiotic stress conditions.

Abiotic stress factors encompass all the environmental vagaries such as drought, salinity, cold and high temperature, which affect the growth and development of a plant (**Figure 2**). These factors are major constraints to crop productivity in semi-arid tropical and subtropical regions, where leguminous crops are predominantly cultivated. Crops grown under abiotic stresses are more susceptible to weeds and biological stress, which directly affect the agricultural productivity. Identification of the molecular mechanisms involved during stress conditions and implementation of this information for improving the quality and yield of plant production would help in developing abiotic stress tolerant crops. There are several studies of differential gene expression under abiotic stress conditions furthering the identification and characterization of candidate resistance gene for the development of abiotic stress tolerant crops.

## Drought stress

Drought is one of the most serious constraints to agricultural productivity that affects plant growth and development. Under drought conditions, normal growth of crops will be negatively affected leading to yield losses or sometimes complete lack of yield under severe conditions. Plants under drought stress experience changes in gene expression patterns to adjust with changed morphological, physiological and metabolic processes to respond to the threatening stress conditions (Cai et al., 2019).

Transcriptomic or differential gene expression studies have become important tools for analyzing drought tolerance mechanism in plants. Differential gene expression study between two or more varieties with significantly different stress tolerance levels is proved to be a very effective strategy for analyzing stress responses in crops (Wang et al., 2016c). In line with transcriptome analysis, Guimarães et al. (2012) performed a large scale screening of drought related candidate genes in a wild peanut and identified several differentially expressed drought related transcription factor genes such as bZIP, MYB, NAC, bHLH and WRKY, while basic leucine zipper (bZIP) was the most abundant among them. In another report, differential gene expression profiles of two peanut wild species were analyzed in response to water deficit, which indicated that drought-responsive genes such as nitrilase, expansin, bZIP and NAC transcription factors were differentially expressed in both genotypes that suggested that these genes played a major role in drought resistance (Brasileiro et al., 2015). Transcriptome analysis of a drought tolerant peanut cultivar variety in response to drought stress at different time points in comparison to control identified several differentially expressed genes including the most abundant LEA family proteins and transcription factors such as MYB, bHLH, WRKY and bZIP. Moreover, differentially expressed genes also were those coding for proteins that are involved in drought stress tolerance, carbohydrate metabolism and photosynthesis (Zhao et al., 2018). Comparative transcriptome study of two wild type peanut species in response to drought stress revealed the genes that are differentially regulated in both genotypes, as the drought tolerant *Arachis duranensis* showed early response to dehydration stress in comparison with the drought sensitive *Arachis stenosperma* (Vinson et al., 2018).

A comparative proteomic and transcriptomic investigation identified several transcripts and proteins associated with drought tolerance that were differentially expressed in the roots of the wild peanut under progressive water deficit. Moreover, in line with the mRNA expression analysis, fifteen identified proteins were similar, but majority of the genes encoding proteins were negatively regulated in stressed roots. Only, a chitinase 2 (Cht2) protein was found to be upregulated in both approaches (Carmo et al., 2019). Wang et al. (2021b) investigated drought induced gene expression in both drought tolerant and susceptible peanut cultivars using a transcriptome profiling study and observed a higher number of differentially expressed genes (DEGs) in the drought-tolerant genotype compared with the drought susceptible cultivar, which were significantly enriched in metabolic pathways, biosynthesis of secondary metabolites and phenylpropanoid biosynthesis. A similar transcriptomic analysis has been carried out in the presence and scarcity of water in peanut cultivars using weighted gene co-expression network analysis (WGCNA) and this investigation revealed that the differentially expressed genes related to transcription factors, carbohydrate metabolism, signal transduction, MAPK signaling pathway, flavonoid and phenylpropanoid biosynthesis are enriched in the drought tolerant peanut cultivar (Zhao et al., 2021). RNA-sequencing technology was also applied to comparative transcriptome analysis of two peanut cultivars in response to drought stress, which found that genes related to ABA and SA signaling were significantly up-regulated. Furthermore, genes related to cell wall hardening, stratum corneum biosynthesis, ROS-scavenging, osmotic-potential and defense-responses were also significantly enriched in favor of tolerance to drought stress (Jiang et al., 2021). Recently, Ren et al. (2022) identified candidate genes associated with drought tolerance by comparative analysis of drought tolerant and drought sensitive cultivated peanut varieties by applying gene regulation network investigations under drought stress. A weighted gene co-expression network analysis (WGCNA) revealed key hub genes related to drought response including genes coding for a potassium transporter, E3 ubiquitin-protein kinase, pentatricopeptide repeat-containing (PPR) protein, protein kinase and aspartic proteinase that were identified under drought stress conditions. WRKY transcription factors play important roles in plant growth and development and response to multiple stresses. RNA-seq study of the drought tolerant peanut genotype, *Arachis duranensis* in response to drought identified several *AdWRKYs*, which were differentially expressed under drought stress. And this study revealed *AdWRKY* gene function and regulatory networks in understanding the phenomenon of drought tolerance in *Arachis duranensis* (Zhang et al., 2022c).

## Salt stress

Soil salinity is another major abiotic stress factor that affects plant growth and development reducing crop productivity. Peanut is considered to be a moderately salt sensitive species, which makes salinity a liming factor for peanut cultivation. In general, plants respond to salt stress by changing their gene expression, which leads to an increase in the concentrations of several metabolites to protect themselves against high salinity. Transcriptome study has become an important tool for studying the possible mechanism and elucidating signal pathways underlying salt stress tolerance in plants. However, limited information is available about the networks of gene expression regulation related to salt stress in peanut. A microarray study has been carried out in peanut roots under salt stress conditions, which revealed that metabolic pathway, biosynthesis of unsaturated fatty acids and plant-pathogen interaction were upregulated, while photosynthesis and phenylalanine metabolism were downregulated (Chen et al., 2016). Transcriptome analysis of cultivated peanut seedlings examined under salt stress treatment revealed that a number of genes were differentially expressed, but two genes encoding a fatty acid desaturase and ω-3 fatty acid desaturase that are involved in the synthesis of linolenic acid were downregulated under salt stress. Interestingly, these were upregulated during recovery from salt stress. Thus, fatty acid desaturase appears to play an important role in salt stress conditions (Sui et al., 2018). Furthermore, the same group has identified a strongly induced gene like tonoplast intrinsic protein 3 (TIP3) under salt stress. Tonoplast intrinsic protein is an aquaporin on the vacuolar membrane that is particularly expressed in seeds under stress, but never reported to be active in shoots and roots (Cui et al., 2018).

A comprehensive study of drought tolerance in cultivated peanut has been performed under salt stress environment and this concurrent stress application has detected several differentially expressed genes and transcription factors (TFs) such as MYB, WRKY, bHLH and AP2/ERF in response to salinity. Moreover, differentially expressed genes related to cell wall growth, antioxidant and peroxidase activity were significantly enriched while DEGs related to metabolic processes, oxidoreductase and catalytic activity were downregulated (Zhang et al., 2020). Aquaporins form a large family of proteins in higher plant playing important roles in balancing water levels in plants under abiotic stress conditions. Han et al. (2021) identified a number of aquaporins in peanut plant under salt stress during differential gene expression analysis and found that an aquaporin protein encoding *TIP3* gene is significantly upregulated in response to salt stress. Furthermore, TIP3 overexpression in *Arabidopsis* resulted in enhanced seed germination under salt stress corroborating its important role in seed germination under salt stress.

Brassinosteroids (BRs) are essential for plant growth and development, and play crucial roles in stress tolerance (Planas-Riverola et al., 2019). However, the role of its exogenous application on the salt tolerance in peanut remains unclear. Recently, Li et al. (2022) found that salt stress inhibits the growth of peanut seedlings and the related transcriptomic study revealed that the exogenous application of 24-epibrassinolide (EBL) upregulated the expression of genes β-fructofuranosidase, sucrose-phosphate synthase 2, *PMP34* that encodes a peroxisomal nicotinamide adenine dinucleotide carrier, and Sodium/H^+^ antiporters (NHX7 and NHX8), and downregulated expression of proline dehydrogenase 2. This resulted in the accumulation of soluble sugars and proline to maintain the osmotic balance with an additional up-regulation of the aquaporin gene *TIP2-1*. Moreover, exogenous EBL application upregulated the expression of *NHX7* and *NHX8* to balance the ion concentrations across membranes with increased peroxidase activity to scavenge reactive oxygen species, and glutathione levels to improve salt tolerance in peanut.

## Cold and metal stress

Low temperature is a major environmental factor that limits plant growth, development and yield. Cold stress causes different degrees of damage to the peanut plant at the seedling, flowering, and all other growth stages. A transcriptome or differential gene expression analysis in response to cold or metal stress could provide a deeper insight into the transcriptional mechanism of plants and their protective role against damage. Differentially expressed peanut genes in response to different temperature regimes was carried out using suppression subtractive hybridization (SSH) for cultivated peanut seeds and this study identified genes that are involved in functional categories including metabolism, defense, stress response, signal transduction and transcriptional regulation (Tang et al., 2011).

Chen et al. (2014a) investigated differentially expressed genes in peanut upon cold stress using the microarray technique and identified the genes involved in biological processes including signal transduction, transcription and translation process, and accumulation of soluble metabolites. Furthermore, their study revealed that the differentially expressed genes are related to protein kinase, heat shock proteins, transcription factors, stilbene synthase and many other protein coding genes, which might play major roles in regulating cold stress in peanut. Differentially expressed proteins of peanut buds in response to cold stress using iTRAQ-based technique were analyzed and a major upregulation of a crucial protein such as Valosin-containing protein (VCP) and many other important pathway proteins related to cold stress were observed (Wang et al., 2020). The transcription factors are essential for the plant to regulate various biological processes. A comparative transcriptome study was carried out in cold-tolerant and sensitive cultivated peanut genotypes to identify transcription factor gene expression under cold stress conditions. Their study identified a number of differentially expressed transcription factors like bHLH, C_2_H_2_, ERF, MYB, NAC and WRKY in response to cold stress, which could be crucial for peanut cold tolerance (Jiang et al., 2020). Transcriptomic analysis of two peanut cultivars (cold tolerant and cold sensitive) at seedlings stage exposed to cold stress revealed a set of cold tolerant genes that are enriched in phenylpropanoid pathway. Moreover, differentially expressed genes involved in soluble sugar, polyamine and G-lignin biosynthetic patrhways were significantly upregulated in the tolerant variety compared to the genotype susceptible to cold stress suggesting their crucial role in peanut at low temperature conditions (Wang et al., 2021a). Another study on transcriptional profiling of two peanut genotypes (cold tolerant and cold sensitive) in response to chilling stress identified several candidate hub genes that appeared to regulate cold tolerance and differentially expressed genes were significantly enriched in pathways related to oxidation-reduction process (Zhang et al., 2022a).

MicroRNAs (miRNAs) are non-coding small RNAs that play important roles in various abiotic stresses by modulating gene expression. However, there is no report on the role of miRNAs in cultivated peanuts during cold stress. Very recently, cold-responsive miRNAs and candidate target genes were identified in peanut cold tolerant and sensitive varieties during cold stress using a deep sequencing method. Their analysis revealed several specific cold responsive microRNAs, which appear to mediate cold response. And, several transcription factors including WDRL, GRF and ARF, and genes such as *DCL*, *SRF* and *SPIRAL* were found to be differentially expressed Suggesting that these might have role in cold tolerance in peanuts (Zhang et al., 2022b).

Aluminum (Al) is the most abundant metal element in the Earth’s crust, and has toxic effects on plant growth in acidic soils (Kochian et al., 2015). The target of aluminium toxicity is the root apex, which reduces root growth and its functions (Horst et al., 2010; Zhu et al., 2014). To overcome the Al toxicity, plants have evolved two resistance mechanisms: one is Al exclusion mechanism, which prevents Al from entering root cells and another one is Al tolerance mechanism, in which Al is sequestrated into vacuoles (Liu et al., 2014; Kochian et al., 2015). However, the molecular regulatory mechanisms of Al toxicity remains unknown in peanut plants. In such a situation, it is pertinent to understand the molecular mechanism of Al toxicity on peanut growth and develop tolerant variety. A RNA-seq based trancriptome study has been considered as an ideal option to discover new genes and estimate transcript abundance during stress or toxicity. Root transcriptome analysis of two peanut cultivars (Al-sensitive and Al-tolerant) in response to Al stress identified a number of differentially expressed Al responsive genes and; the DEGs were enriched in pathways related to organic acid transport, transcription regulation, metal cation transport and programmed cell death (PCD). Furthermore, weighted correlation network analysis (WGCNA) identified a highly expressed Al-associated gene *EIL* (*EIN3-like*), which reveals a link between ethylene signal transduction and Al resistance related genes in peanut (Xiao et al., 2021). An ultrasonic treatment efficiently improved crop tolerance to metal toxicity, but ultrasonic induced aluminium (Al) tolerance is poorly studied in peanuts. Recently, a transcriptomic study of utrasonic seed treatment of peanut revealed that transcription factors such as AP2, bHLH, WRKY, MYB and NAC, and plant hormone pathways namely SA, ABA, IAA and JA were significantly upregulated in ultrasonic treated peanut seeds in comparison to control seeds. Moreover, these significantly induced genes of plant hormone signal transduction and transcription factors play important roles in Al tolerance in peanuts (Bao et al., 2022b). The same group also showed that Al treatment at low concentration elevates peanut growth, while high concentrations of Al significantly reduced peanut plant growth. In comparison to control, a trancriptomic study of Al treated plants showed differentially expressed genes (DEGs) of sucrose and starch metabolic pathways that were significantly upregulated at low concentrations of Aluminium. Interestingly, the plant hormone signaling pathway was significantly upregulated at higher concentration of Aluminium treatments. Furthermore, the expression of transcription factors (TFs) in peanut is concentration dependent and overall, this study showed that Al at low concentration acts as a stimulant, while at high concentration it is toxic and acted as an inhibitor (Bao et al., 2022a).

Differential gene expression study of peanut plants in response to abiotic stresses, reported by the most of the studies (**Table 3**), the commonly expressed genes are transcription factors (MYB, WRKY, NAC, bZIP, bHLH, AP2/ERF, C3H, DREB, MAD-box), LEA proteins, chitinase, aquaporins, F-box protein, cytochrome p450, zinc finger, peroxidase, superoxide dismutase, fatty acid desaturase, protein phosphatase, protein kinases and transferases. These differentially expressed genes are involved in the process or enriched pathway of transcription regulations, starch and sucrose metabolism, signal transduction, metabolic process, photosynthesis-antenna proteins and biosynthesis of unsaturated fatty acids. Some of these differentially expressed genes were successfully incorporated in peanut or model plants (tobacco or Arabidopsis) through genetic manipulation to develop abiotic stress tolerant plants (**Table 4**). These differentially expressed genes in *Arachis* accessions under drought, salt, cold and metal stress conditions will provide a better understanding of the tolerance mechanism, and will further provide reference for improving abiotic tolerant peanut cultivar through genetic manipulation.

**Table 3:** Some important differentially expressed peanut genes and enriched pathways or genes involved in process during various abiotic stress conditions.

Stresses	Technique used	Some important differentially expressed genes (DEGs)	Enriched pathways/genes involved in	References
Drought	RNA-seq	transcription factors (bZIP, MYB, NAC, bHLH, AP2-EREB, WRKY), R gene (NBS-LRR), serine/threonine protein kinases, peroxidases, catalases, chitinases, glycosinases	transcriotion factors, defense related, metabolic process	Guimarães et al. (2012)
	SSH	transcription factors (bZIP, NAC), cytochrome p450, carbonic anhydrase, metallothioneins, chloroplast drought-induced stress protein (CDSP), expansin-like B, nitrilase, drought-induced proteins	transcription factors, metabolic process, cellular process, response to abiotic stimulus	Brasileiro et al. (2015)
	RNA-seq	transcription factors (MYB, bHLH, bZIP, WRKY, ERF), chalcone synthase, chalcone isomerase, F-box protein, LEA protein (LEA2, LEA3, LEA4), cytochrome, peroxidase, dihydroflavonol 4-reductase, glycerol-3-phosphate dehydrogenase	transcription factors, photosynthesis, carbon metabolism, citrate cycle	Zhao et al. (2018)
	RNA-seq	transcription factors (MYC, MYB, bZIP, NAC, WRKY, and DREB), galactinol synthase, asparagene synthetase, ABA hydroxilase, dehydrin, cysteine protease, expansin, aquaporins, F-box protein, protein kinases (kinase and mitogen), ACC synthase	transcription regulation, starch and sucrose metabolism, dehydration response, signaling	Vinson et al. (2018)
	2D/RNA-seq	chitinase-2, heat shock protein (HSP70), MLP-like protein, maturase K, glycine-rich protein DOT1-like, adenine phosphoribosyl transferase, transcription initiation factor IIF subunit alpha-like (TFIIF)	response to abiotic stimulus, RNA processing, unassigned function	Carmo et al. (2019)
	RNA-seq	transcription factors (bHLH, NAC, WRKY), chitinase, protein kinase, protein phosphatase, caffeoyl-CoA 3-O-methyltransferase, peroxidase, glutathione S-transferase, cinnamyl alcohol dehydrogenase, glutathione reductase, dehydroascorbate reductase, beta glucosidase, auxin response factor, SAUR-like auxin-responsive protein	transcription factors & MAPK signaling, flavonoid biosynthesis, phenylpropanoid biosynthesis, starch and sucrose metabolism, signal transduction	Zhao et al. (2021)
	RNA-seq	transcription factor (bZIP, bHLH, MYB, ERF, NAC), transferase, stilbene synthase, glutathione S-transferase, F-box protein, ABA 8’-hydroxylase, protein kinase, serine/threonine protein kinase, chalcone synthase, chalcone isomerase, F-box protein, phosphatase, LEA protein, heat shock protein, cyclophilin	metabolic process, secondary metabolism, plant-circadian rhythm, phenylpropanoid biosynthesis, starch and sucrose metabolism	Wang et al. (2021b)
	RNA-seq	LRR treceptor like protein kinase, serine/threonine protein kinase, F-box protein, pentatricopeptide repeat-containing protein, catalase, potassium transporter, E3 ubiquitin-protein ligase, ribokinase, aspartic proteinase, monooxygenase, cysteine synthase, aminopeptidase, brassinosteroid insensitive 1-associated receptor kinase	carbon metabolism, photosynthesis pathway, phenylalanine metabolism, galactose metabolism, sphingolipid metabolism	Ren et al. (2022)
Salt	Microarray	transcription factors (MYB, WRKY, AP2/ERF, NAC, bZIP, bHLH), zinc finger, superoxide dismutase, amine oxidase, catalase, ascorbate peroxidase, cinnamate 4-hydroxylase, glucosyl transferase, glycoside hydrolase, acid phosphatase, PERK1-like protein kinase, UDP-glucosyltransferase, glutathione peroxidase, thioredoxin	transcription regulation, metabolic pathway, biosynthesis of unsaturated fatty acid, photosynthesis, phenylalanine metabolism	Chen et al. (2016)
	RNA-seq	acyl-CoA synthetase, alcohol dehydrogenase, fatty acid desaturase, peroxisomal 3-ketoacyl-CoA thiolase, 4-coumarate-CoA ligase, 12-oxo-phytodienoate reductase, glucose 1-dehydrogenase, serine-type endopeptidase, retinol dehydrogenase, oxidoreductase	fatty acid metabolism, biosynthesis of unsaturated fatty acid, linolenic and linoleic acid metabolism, fatty acid biosynthesis	Sui et al. (2018)
	RNA-seq	LEA proteins, K+ transporter, aquaporins, Na+ transporters, Na+/H+ antiporter, H+-pyrophosphatase, ascorbate peroxidase, superoxide dismutases, catalases, peroxidases, glutathione S-transferases, peroxiredoxins, alternative oxidases, photosystem I & II, rubisco, proline dehydrogenase	metabolic pathway, biosynthesis of secondary metabolites, ether lipid metabolism, photosynthesis-antenna proteins	Cui et al. (2018)
	RNA-seq	transcription factors (MYB, AP2/ERF, WRKY, bHLH, bZIP, HSF, MADS-box), chalcone synthase, chalcone-flavanone isomerase, cinnamyl alcohol dehydrogenase, phenylalanine ammonia lyase, nitrate reductase, glutathione S-transferase, peroxidase, catalase, superoxide dismutase, V-ATPase, V-Ppase, K+ transporter family protein, F-box, zinc finger, cytochrome p450	transcription factors, phenylpropanoid biosynthesis, starch and sucrose metabolism, plant circadian rhythm, flavonoid biosynthesis	Zhang et al. (2020)
	RNA-seq	peroxisomal nicotinamide adenine dinucleotide carrier, beta-fructofuranosidase, probable sucrose-phosphate synthase, Na+/H+ antiporters, peroxidase, enoyl-CoA hydratase, cytochrome p450, BR signal kinase, aquaporin (TIP2-1), proline dehydrogenase, dehydrins, proline-rich receptor-like kinase, catalase, ethylene responsive transcription factor, peroxiredoxin, L-ascorbate peroxidase	photosynthesis-antenna proteins, starch and glucose metabolism, signal transduction, alanine, aspartate and glutamate metabolism	Li et al. (2022)
Cold	SSH	transcription factors (NAC, MYB), zinc finger, glutathione S-transferase, LEA protein, heat shock protein, cyclophilin, metallothionein, F-box protein, protein phosphatase, galactosyltransferase, malate dehydrogenase, defensin, aldehyde dehydrogenase, vacuolar-processing enzyme, sgt1-like protein	transcription regulation, metabolism, signal transduction, stress and defense related	Tang et al. (2011)
	Microarray	transcription factors (MYB, WRKY, NAC, bZIP, bHLH, AP2/ERF), fatty acid desaturase, sphingolipid desaturase, acetyl-CoA carboxylase, aldolase, stilbene synthase, desiccation-related protein, glucosidase, glucanase, fructofuranosidase, peroxidase, LEA protein, NBS-LRR protein, heat shock protein, chitinase, MAP kinase, protein phosphatases, protein kinases, CBL interacting protein kinase	transcription regulation, transport process, carbohydrate biosynthesis, metabolic process, signal transduction	Chen et al. (2014a)
	RNA-seq	transcription factors (bHLH, MYB, C2H2, NAC, WRKY, bZIP, ERF, C3H), CDP-diacylglycerol-inositol 3-phosphatidyltransferase, phospholipase D, diacylglycerol acyltransferase, lipoxygenase, 3-ketoacyl-CoA synthase, protein phosphatase, receptor like protein, serine/threonine-protein kinase, WD40 repeat-like-protein, mitogen-activated protein kinase, defensin-like protein, isochorismate synthase	transcription regulation, signal transduction, plant-pathogen interaction, MAPK signaling	Jiang et al. (2020)
	RNA-seq	transcription factors (MYB, WRKY, C2H2, bHLH, bZIP, zinc finger), chalcone synthase, 4-coumarate-CoA ligase, phenylalanine ammonia lyase, dehydrin, 6-phosphogluconate dehydrogenase, pentatricopeptide repeat family protein, F-box protein, cytochrome p450, galactinol synthase, raffinose synthase, spermidine synthase, calcium-dependent protein kinase, heat shock transcription factors, superoxide dismutase, peroxidase, protein phosphatase	transcription regulation, phenylpropanoid biosynthesis, linoleic acid metabolism, stelbenoid biosynthesis, gingerol biosynthesis	Wang et al. (2021a)
	RNA-seq	transcription factors (NAC, bHLH), zinc finger protein, protein kinase, receptor-like protein kinase, catalase, allene oxide synthase, E3 ubiquitin-protein ligase, protein phosphatase 2C, glutathione S-transferase, polyamine oxidase, catalase, peroxidase, superoxide dismutase, ascorbate peroxidase	transcription regulation, oxidation-reduction process, protein phosphorylation, carbohydrate metabolism	Zhang et al. (2022a)
Metal	RNA-seq	transcription factors (AP2-EREBP, WRKY, bHLH, NAC, MYB, C2H2), cytochrome p450, citrate transporters, Al-activated malate transport, aluminium sensitive 1, pectin methylesterase, xyloglucan endotransglucosylase, malate dehydrogenase, respiratory burst oxidase, metacaspase-3-like gene, ethylene biosynthetic genes, EIL (EIN3-like) gene, NADPH oxidases, multidrug and toxin extrusion	transcription regulation, organic acid and metal cation transport, carbohydrate metabolic process, photosynthesis-antenna proteins, plant-pathogen interaction	Xiao et al. (2021)
	RNA-seq	transcription factors (MYB, bHLH, NAC, ASR, STOP1, ABI5, RAE, WRKY), aluminum-activated malate transporter 1, F-box protein, sugar transporter (ERD6), ferric reductase defective like 1, abscissic acid and stress ripening (ASR) gene, 9-cis-epoxycarotenoid dioxygenase, MADS-box transcription factor, ADP-ribosyltransferase 1, C2H2 zinc finger protein	transcription regulation, starch and sucrose metabolism, phenylpropanoid biosynthesis, signal transduction, plant-pathogen interaction	Bao et al. (2022b)
	RNA-seq	transcription factors (AP2, WRKY, bHLH, bZIP, NAC, MYB), ADP-ribosyltransferase 1, malate transporter, ferric reductase defective like 1(FRDL1), F-box protein, auxin-responsive transcription factor (ARF), glutathione S-transferase, aluminum-activated malate transporter 1, peroxidase, catalase, ascorbate peroxidase, cinnamate 4-hydroxylase, glucosyl transferase	transcription regulation, peroxisome and endocytosis, signal transduction, phenypropanoid biosynthesis, starch and sucrose metabolism	Bao et al. (2022a)

**Table 4:** List of differentially expressed peanut genes used in enhanced abiotic tolerance.

Gene	Characterized in	Stress tolerance	Identification method	Promoter used	References
*AhERF019*	*Arabidopsis*	Drought, heat and salt tolerance	EST sequences	CaMV35S	Wan et al. (2014)
*AdLEA*	tobacco	abiotic stress	cDNA-AFLP	CaMV35S	Sharma et al. (2016)
*AdCIPK5*	tobacco	abiotic (salt & osmotic) stress	Genefishing DEG	CaMV35S	Singh et al. (2020)
*AdGolS3*	*Arabidopsis*	Abiotic stress tolerance	RNA-seq	CaMV35S	Vinson et al. (2020)
*AhTIP3;1*	*Arabidopsis*	Salt tolerance	RNA-seq	CaMV35S	Han et al. (2021)
*AhWRKY75*	peanut	Salt tolerance	RNA-seq	CaMV35S	Zhu et al. (2021)
*AhbHLH112*	*Arabidopsis*	Abiotic (drought) stress	RNA-seq	CaMV35S	Li et al. (2021)
*AhCytb6*	tobacco	abiotic (N2-starvation & salt) stress	suppression subtractive hybridization (SSH)	CaMV35S	Alexander et al. (2021)
*AhBINR*	tobacco	abiotic (N2-starvation & salt) stress	Microarray	CaMV35S	Alexander et al. (2022)
*AhLOX29*	*Arabidopsis*	Abiotic (drought) stress	RNA-seq	CaMV35S	Mou et al. (2022)
*AhSAUR3*	*Arabidopsis*	Drought tolerance	RNA-seq	CaMV35S	Liu et al. (2022)

## Transgenic plants expressing differentially expressed peanut genes for enhanced abiotic stress tolerance

Further characterization of differentially expressed genes during various abiotic stresses by genetic manipulation like transgenic approach would aid in developing stress tolerance peanut cultivars, which can help achieve increased crop productivity. Till today, different types of transgenic plants were generated by genetic engineering technique using *Agrobacterium tumefaciens* mediated genetic transformation. There are several reports on the characterization of differentially expressed genes in peanut and/or model plants to develop stress resistance plants. The list of transgenic plants deploying differentially expressed peanut genes is provided in **Table 4**.

Ethylene-responsive factor (ERF) plays a significant role in regulating gene expression in plant responses to stresses and was identified from peanut EST sequences available in the NCBI database. Ectopic expression of *AhERF019* in *Arabidopsis* resulted in enhanced tolerance to drought, heat, and salt stresses (Wan et al., 2014). Late Embryogenesis Abundant (*LEA*) gene was differentially expressed in wild peanut upon infection with late leaf spot pathogen and overexpression of *AdLEA* in tobacco resulted in enhanced tolerance of plants to dehydration, salinity and oxidative stress. Furthermore, *AdLEA* overexpressed tobacco plants maintained better photosynthetic efficiency under drought conditions implying that it could be a potential gene for genetic modification in crop plants (Sharma et al., 2016). *In silico* analysis of RNA-seq data of wild peanut *Arachis duranensis* in response to drought stress identified a galactinol synthase (*GolS3*) gene and overexpression of *AdGolS3* gene in *Arabidopsis* resulted in increased raffinose production and tolerance to drought, salt and osmotic stresses (Vinson et al., 2020). A CBL-interacting protein kinase (CIPK5) was differentially expressed in wild peanut upon challenge with fungal infection and *AdCIPK5* overexpressed tobacco plants displayed NaCl and osmotic tolerance, again indicating that it could be a novel gene for abiotic stress tolerance in plants (Singh et al., 2020).

Aquaporins play a crucial role in seed germination (Maurel et al., 2015) and an aquaporin isoform *TIP3* was found differentially upregulated under salt stress condition in cultivated peanut. Furthermore, the ectopic expression of *AhTIP3;1* contributed to improved seed germination under salt stress in Arabidopsis (Han et al., 2021). WRKY transcription factors are involved in plant growth and development, defense and stress responses (Ning et al., 2017). A novel WRKY transcription factor family gene, *AhWRKY75* was isolated from salt-tolerant mutant M34, which was differentially upregulated in response to salt stress. The constitutive overexpression of *AhWRKY75* enhanced tolerance to salt stress by improving ROS scavenging system and photosynthetic efficiency in peanut (Zhu et al., 2021). A differentially expressed clone SM409 (that has similarity with *Cytb6*) was found upregulated during plant-microbe interaction in peanut and further, the overexpression of *AhCytb6* gene in tobacco resulted in enhanced seed germination under N_2_ deficit and salt stress conditions (Alexander et al., 2021).

Transcriptome analysis of cultivated peanut in response to drought stress identified some transcription factor *bHLHs* genes as differentially expressed, and these included *AhbHLH112* (Zhao et al., 2018). Overexpression of *AhbHLH112* in *Arabidopsis* enhanced drought tolerance both in seedlings as well as at adult stages by regulating ROS scavenging pathways to protect the plants against drought stress (Li et al., 2021). Lipoxygenases (LOXs) belong to a family of proteins that play important roles in plant development and defense responses. Differential expression patterns of peanut *LOX* genes were analyzed under drought and salt stresses using published RNA-seq results and it was shown that *AhLOX29* was strongly upregulated in response to abiotic stress condition. Ectopic expression of *AhLOX29* in *Arabidopsis* resulted in enhanced tolerance to drought stress (Mou et al., 2022).

In a microarray study, a novel gene *AhBINR* was differentially upregulated during interaction between cultivated peanut and *Brachybacterium saurashtrense* under nitrogen starvation conditions and the overexpression of *AhBINR* gene exhibited high photosynthetic efficiency with increased tolerance to salt stress and nitrogen deficit conditions in transgenic tobacco (Alexander et al., 2022). Small auxin-up-regulated RNAs (SAURs) gene family plays major roles in plant growth, development, and stress responses. Another differential expression profiling study revealed that SAUR genes were dominantly expressed in most of the vegetative parts of the plant and appear to be involved in abiotic stress tolerance, while overexpression of *AhSAUR3* showed decreased tolerance to drought stress in *Arabidopsis* (Liu et al., 2022).

## Transgenic plants expressing peanut genes identified from DEGs for enhanced biotic and abiotic stress tolerance

Some of the peanut genes, which were differentially expressed during abiotic or biotic stress conditions are further characterized by generating transgenic plants. These genes were further characterized in model plants like tobacco or *Arabidopsis*, and they exhibited enhanced tolerance to both abiotic and biotic stresses (**Table 5**). Thaumatin-like (PR-5) proteins identified in differential expression study of a wild peanut showed enhanced tolerance to both biotic and abiotic stress conditions in tobacco (Datta et al., 1999). In the differential gene expressions study using *Arachis diogoi* treated with the late leaf spot pathogen, a thaumatin like protein (*AdTLP*) gene was observed to be upregulated (Kumar and Kirti, 2011; Kumar and Kirti, 2015b). Overexpression of *AdTLP* in tobacco plants exhibited enhanced resistance to the fungal pathogen, *Rhizoctonia solani* and the transgenic seedlings also exhibited enhanced tolerance against salt and oxidative stress. Moreover, purified recombinant thaumatin like protein showed enhanced antifungal actvity and these results suggested that the *AdTLP* could be a good candidate gene for enhancing stress resistance in crop plants (Singh et al., 2013).

**Table 5:** List of differentially expressed peanut genes used to enhanced biotic and abiotic tolerance.

Gene	Characterized in	Stress tolerance	Identification method	Promoter used	References
*AdTLP*	tobacco	biotic and abiotic stresses	Genefishing DEG	CaMV35S	Singh et al. (2013)
*AdDjSKI*	tobacco	biotic and abiotic stress	cDNA-AFLP	CaMV35S	Rampuria et al. (2018)
*AdDHN1*	*Arabidopsis*	drought tolerance but susceptible to root knot nematode (RKN)	*In silico* EST	Arabidopsis actin 2 promoter (ACT-2)	Mota et al. (2019)
*AhGLK1b*	*Arabidopsis*	biotic (fungal & bacterial) and abiotic stress	Microarray	CaMV35S	Ali et al. (2020)
*AdEXLB8*	tobacco	biotic and abiotic stress	RNA-seq	CaMV35S	Brasileiro et al. (2021)

A gene encoding a serine-rich DnaJIII protein (*AdDjSKI*) was found to be differentially expressed in the wild peanut after late leaf spot infection (Kumar and Kirti, 2015b). Overexpression of *AdDjSKI* conferred tolerance to multiple stresses like heat, salinity, drought and osmotic, along with enhanced resistance to *Phytophthora parasitica* pv *nicotiana*e and *Sclerotinia sclerotiorum* in tobacco through ectopic expression (Rampuria et al., 2018). Plant dehydrins (DNHs) belong to the LEA protein family involved in responses to multiple abiotic stresses and *AdDHN1* was identified in a wild peanut by *in silico* expression patterns of DHNs gene. Overexpression of *AdDHN1* in *Arabidopsis* displayed improved tolerance to cold and drought, but increased susceptibility to the biotrophic parasite root-knot nematode (Mota et al., 2019). The GOLDEN2-LIKE (GLK) transcription factor (TF) is a member of the myeloblastosis (MYB) family and plays a significant role in the regulation of plastid biogenesis and stress tolerance (Li et al., 2018). *AhGLK1b* was identified from a cultivated peanut showing down-regulation in response to low calcium level during a microarray analysis (Chen et al., 2016). Ectopic expression of *AhGLK1b* resulted in enhanced resistance to phytopathogen *Sclerotinia sclerotiorum* and bacterial pathogen *Pseudomonas* Pst DC3000 and also increased tolerance to abiotic stresses in *Arabidopsis* (Ali et al., 2020).

Plant expansins are cell wall loosening proteins implicated in various developmental activities and responses to both abiotic and biotic stresses (Cosgrove, 2015; Marowa et al., 2016). Recently, an expansin like B (EXLB) gene was identified as significantly upregulated in a wild peanut in response to multiple stress treatments (Mota et al., 2021). Furthermore, overexpression of *AdEXLB8* resulted in enhanced tolerance to drought stress and biotic (*Sclerotinia sclerotiorum* and *Meloidogyne incognita*) stress in tobacco (Brasileiro et al., 2021).

## Era of microRNAs studies in peanut

MicroRNAs (miRNAs) are important endogenous non-coding RNAs with an average size of 21 to 23-nt in length that regulate gene expression in plants and animals by regulating mRNA expression post-transcriptionally (Ambros, 2004; Zhang et al., 2006; Zhang et al., 2009). RNA polymerase II transcribes miRNA from miRNA genes, which are mainly located in the intergenic regions of the genome (Moss and Poethig, 2002; Kim, 2005). Plant miRNAs regulate gene expression by complementing target genes completely and further excise the target genes from the genome to inhibit gene expression. The target genes mainly include transcription factors, enzymes, signaling proteins etc. MicroRNAs have been shown to be involved in various biological and metabolic processes including plant growth, development and response to environmental stresses (Reinhart et al., 2002; Jones-Rhoades et al., 2006). The miRNAs play key roles in plant responses to the environment and were up- and down-regulated by abiotic stresses including drought, salinity and chilling (Sunkar and Zhu, 2004; Liu et al., 2008). There were extensive studies toward identifying miRNAs and analyzing their functional role in response to biotic stresses in various plant species including crops (Jagadeeswaran et al., 2009; Padmanabhan et al., 2009; Zhang et al., 2011; Feng et al., 2014; Li et al., 2014).

However, there were limited reports on the identification of miRNAs in peanut due to unavailabilty of complete genome sequence till recently. However, in line with miRNAs studies in peanut, Zhao et al. (2010) reported 14 novel and 75 conserved miRNAs that might play crucial roles in plant growth, development and environmental stresses using deep sequencing. A high-throughput sequencing method of peanut small RNA library identified a large number of miRNAs and their related target genes (Chi et al., 2011). Despite these earlier studies, the focus on the role of miRNAs in peanut for growth and developmental process is still limited (Ma et al., 2018; Figueredo et al., 2020). The knowledge regarding the miRNAs expression in peanut under stress conditions are further limited. Recently, an integrated analysis of transcriptome and small RNAs revealed potential miRNAs and their target genes (mRNA) pairs in susceptible and resistant peanut seeds in response to *Aspergillus flavus* (Zhao et al., 2020).

For identification of key regulatory miRNA-targets that regulate programmed cell death (PCD) under Al stress, root tips of Al-sensitive and Al-tolerant peanut cultivars were analyzed under Al stress condition. Further intergrated analysis of transcriptomics, sRNAs, and degradome data sets revealed differential expression of 89 miRNA-mRNA interactions that might be involved in PCD under Al stress (Tong et al., 2022). In response to cold stress several cold-responsive miRNAs and their target genes were identified through integrated analysis of small RNA and degradome in peanut sensitive and tolerant lines during stress condition (Zhang et al., 2022b). Thus, a systematic study of differential gene expression (DEGs) in peanut under stress conditions, transcriptome analysis along with miRNA/sRNA and degradome study would provide a more precise and reliable gene expression analysis and their regulation.

## New era of genome editing in peanut

The biological stresses mainly comprise attack from pathogenic fungi, bacteria, viruses, nematode and insects, whereas the abiotic constraints include drought, salinity, cold, metal, waterlogging and temperature changes. To overcome these various stress environments, plants must be capable of tolerating these stress conditions by modulating their metabolism in the right direction. In this direction, the genetically modified crops are considered to be good candidates for sustainable food production. It is to be noted that there were several genes identified in *Arachis* species using differential expression analysis for stress tolerance. However, commercially cultivated transgenic plants were not developed in peanut so far using the identified genes. There could be several reasons for this. Since peanut is a crop of high commercial significance whose produce is of prime importance in human nutrition, it is not easy to get regulatory approvals from the respective Governmental authorities across the world for the deployment of transgenic plants for field- level cultivation. Probably, many major efforts were not made in peanut in this direction because of this reason. With the advent of genome editing technology for various plant systems, the future is very optimistic for developing genome edited crops including peanut for stress tolerance as genome-edited crops might get regulatory approvals more easily compared to transgenic plants.

Genome modifications allow improvements in stress and weed tolerance, plant breeding procedure, productivity, food quality and safety. However, introduction of genetically modified crops may adversely affect the environmental conditions exercising harmful effects on animals and humans and this has been the main concern of the environmentalists (Aziz et al., 2022). There are several strategies to transfer gene of interest into the plant genome. The most effective and universal method of desired gene incorporation into the plant genome is the *Agrobacterium tumefaciens* or *Rhizobium rhizogenes* mediated genetic transformation technique through either *in vitro* or *in planta* methods for improvement. Transgene expression is highly dependent on the promoter selection for gene expression followed by protein synthesis. Several promoters of various origins have been tested for transgene expression besides viral gene promoter *CaMV35S* in peanut. In the backdrop of this, no meaningful progress of gene editing technique that is gaining enormous attention recently has been made so far in peanut.

There are several powerful plant-targeted genome editing tools for functional and applied genetic manipulations that include engineered nucleases, such as Zinc finger nucleases (ZFNs), transcription activator-like effector nucleases (TALENs) and clustered regulatory interspaced short palindromic repeats (CRISPR) systems (Lloyd et al., 2005; Cermak et al., 2011; Mahfouz et al., 2011; Li et al., 2012; Jiang et al., 2013; Puchta, 2017; Bhardwaj and Nain, 2021).

Recently developed prokaryotic immune system based CRISPR technology is a high-throughput genome editing tool that has been found to be very successful in a variety of plant species (Pandey et al., 2019; Tiwari et al., 2021). The CRISPR system is based on the RNA-guided interference (RNAi) with DNA, which can create multigene deletion, traslocation or additions in chromosome (Koonin et al., 2017; Salsman and Dellaire, 2017). This gene manipulation technique emerged as a powerful alternative to plant breeding due to its efficient, precise and targeted gene modification which results in rapid improvement of crops (Nekrasov et al., 2013). CRISPR/Cas9 based genome editing has been used for developing abiotic and biotic stress tolerance in crop plants for sustainable food production (Aziz et al., 2022). In Tomato, this technique has been utilized for developing drought, salt, chilling and heat stress tolerance (Wang et al., 2017; Yin et al., 2018; Tran et al., 2021), while, it was used in enhancing drought, temperature and metal tolerance in rice (Tang et al., 2017; Wang et al., 2017; Miao et al., 2018; Wen et al., 2021).

Biotic stress tolerance where crop yield and quality are largely affected by biotic stresses like viral, fungal, bacterial and insects were improved by using CRISPSR/Cas9 system. For instance, wheat and rice have been made tolerant to their respective viral, bacterial and fungal diseases (Wang et al., 2014; Wang et al., 2016a; Macovei et al., 2018). Tomato, which is largely affected by various fungal, viral and bacterial pathogens, has been made resistant to these pathogens via the CRISPR/Cas9 application (Paula De Toledo Thomazella et al., 2016; Nekrasov et al., 2017).

Application of CRISPR/Cas9-based gene editing technique would provide genetic improvement in peanut cultivars against biotic and abiotic stresses. However, application of this breakthrough gene editing technology to peanut is still rare. Till date, there is limited report of CRISPAR/Cas9-based gene editing in peanut. This technique is used for knocking out fatty acid desaturase2 (*FAD2*) gene in peanut. This gene codes for the desaturase that is responsible for the conversion of monounsaturated oleic acid into polyunsaturated linoleic acid. The knock out mutation in *FAD2* resulted in high oleic acid content in peanut oil (Yuan et al., 2019; Neelakandan et al., 2022). Shu et al. (2020) showed that CRISPR/Cas9-based gene editing of peanut *AhNFR* gene through hairy root transformation system and validated the function for nodule formation in peanut. Using the CRISPR/Cas9 system, *AhFatB* genes *Arahy4E7QKU* and *ArahyL4EP3N* were knocked out in peanut, and mutation at *Arahy.4E7QKU* displayed low palmitic acid and high oleic acid content significantly improving oil quality in peanut (Tang et al., 2022). Potential application of CRISPR/Cas9 mediated genome editing system in peanut would be the best stratégy to manipulate taits in peanut, which can leads to improvements in biotic and abiotic stress tolerance, nutritional quality such as oil quality and allergen, and further help in generating new peanut genotypes that might be useful in peanut breeding programs.

## Future perspectives

The wild germplasm belonging to the genus *Arachis* divided into various Sections is a boon to the investigators undertaking differential expression studies using the previously popular techniques like cDNA-AFLP and the highly efficient RNA-Seq studies using the Next Generation Sequencing technologies that became available recently. The goal of these studies is to understand molecular mechanisms during stress conditions and identify the genes responsible for stress tolerance in peanut plants. Depending on the availability of financial resources, the choice of the technology can be made. The primary advantage with the NGS technologies is that they give faster results on gene expression at whole genome level compared to the earlier technologies. The wild *Arachis* germplasm offers excellent material for the differential expression studies because of the availability of genotypes that are both susceptible and highly resistant to corresponding trait, be it biotic or abiotic stress tolerance. The alloploid species belonging to the Section *Rhizomataceae* are very highly tolerant to abioic stresses like high temperature and water limited conditions. Hence, the germplasm can be used for identifying tolerance genes within the tolerant genotype itself by comparing sets with and without treatments. Otherwise, a more efficient option would be to compare the resistant and susceptible genotypes at the same ploidy level with and without suitable treatments. Information from such studies could lead to novel genes that would actually decide the trait under consideration. The genes identified can be used in other related legume crop plants. This is particularly because of the possible colinearity of different related genomes in legumes. Hence, the opportunities are enormous when the investigator uses the material judiciously.

## Author contributions

DK: Conceptualization, Writing – original draft. PBK: Conceptualization, Writing – review & editing.
